# *Primulina malipoensis* (Gesneriaceae), a new species from Sino-Vietnamese border area

**DOI:** 10.3897/phytokeys.94.20861

**Published:** 2018-01-29

**Authors:** Li-Hua Yang, Jun-Lin Chen, Fang Wen, Ming Kang

**Affiliations:** 1 Key Laboratory of Plant Resources Conservation and Sustainable Utilisation, South China Botanical Garden, Chinese Academy of Sciences, Guangzhou 510650, China; 2 University of Chinese Academy of Sciences, Beijing 100049, China; 3 College of Humanities Sichuan Agricultural University, Ya’an, Sichuan 625014, China; 4 Gesneriad Conservation Centre of China, Guangxi Institute of Botany, Guangxi Zhuang Autonomous Region and Chinese Academy of Sciences, Guilin 541006, China

**Keywords:** Limestone flora, New taxon, Sino-Vietnamese border area, Taxonomy

## Abstract

*Primulina malipoensis*, a new species from limestone areas around the Sino-Vietnamese border, is described and illustrated. This new species is morphologically similar to *P. maguanensis* and *P. lungzhouensis*, but obviously differs from the latter two species by its pale greenish-yellow flowers (vs. purple, with different colour patterns). The phylogenetic affinity, illustration and photographs of this new species are provided in this paper.

## Introduction

The recently redefined *Primulina* Hance has become a species-rich genus within the subfamily Didymocarpoideae of Gesneriaceae (Wang et al. 2011, Weber et al. 2011, 2013) and its species diversity is still growing due to numerous new species being constantly discovered (e.g. Pan et al. 2013, Guo et al. 2015, Lai and Wen 2015). This group shows high levels of endemism and ecological (edaphic) specialisation (Hao et al. 2015). The majority of its species occur in karst areas of southern and southwestern China and northern Vietnam, with narrow, island distributions, often limited to a single cave or karst limestone hill system (Wang et al. 1998, Li and Wang 2004, Wei et al. 2010). Local-scale mosaics of soil type are ubiquitous features in the karst landscapes and thus, soil nutrient availability may influence diversification and speciation of *Primulina* via local adaptation to specific edaphic microhabitats (Hao et al. 2015). However, in the *P. eburnea* complex, geographical isolation has been shown to be a major driver of its diversification and speciation in *Primulina* (Gao et al. 2015, Wang et al. 2017).

During field explorations in 2013, one of the authors (JC) found an unknown species of *Primulina* near the Sino-Vietnamese border at Malipo County, southeastern Yunnan, China. Several living individuals from the population found in the field were brought to the South China Botanical Garden (SCBG) and cultivated there. These plants showed leaf blade characteristics very common in *Primulina*. However, when flowering, they displayed uncommon yellow flowers. Flower colour has been used as an important character for the description of new *Primulina* species (Pan et al. 2016, Yang et al. 2017). Therefore, these plants soon caught the authors’ attention. Checking of specimens and literature studies were undertaken immediately. When specimens were checked in KUN (by its online service), an interesting specimen was found (numbered *KUN 1275938*), which possesses a similar leaf to these plants and had been collected from nearly the same locality as the findings. This specimen was identified as *Chirita eburnea* (a synonym to *P. eburnea*). However, this specimen was represented by only a piece of leaf and without flowers, thus, its identification is doubtful. To further reveal the true taxonomic identity of both of these plants and the specimen, other field works were carried out by one of the authors (FW) in 2017. Fortunately, he found this species at the recoded site of the specimen (*KUN 1275938*) and also found other populations at a nearby location in Vietnam. At the same time, additional investigations, i.e. phylogenetic analysis and morphological comparison, were undertaken. Based on these results, all of these plants from the three populations are considered as the same new species, which is described and illustrated here.

## Methods

Morphological observations were carried out using living cultivated plants (ten individuals) as well as dried specimens. All morphological characters were measured using dissecting microscopes and descriptions were made following the terminology presented by Wang et al. (1998). Literature studies included all relevant monographs (Wang et al. 1998, Li and Wang 2004, Wei et al. 2010) and recently published literature (Xu et al. 2008, 2012, Li and Möller 2009, Pan et al. 2013, 2016, Li et al. 2014, Lai and Wen 2015, Guo et al. 2015). Checking of specimens was undertaken at IBSC and IBK and with the help of web databases (Chinese Virtual Herbarium: http://www.cvh.ac.cn/; Herbarium, Kunming Institute of Botany, CAS: http://www.kun.ac.cn/; Global Plants: http://plants.jstor.org/). A map of the species’ geographical distribution was prepared based on field records. The molecular phylogenetic analyses of the species were included in a broader study in which the most comprehensive species-level phylogeny of *Primulina* was reconstructed based on 20 plastid and nuclear regions (Kong et al. 2017).

## Taxonomy

### Primulina malipoensis

Taxon classificationPlantaeLamialesGesneriaceae

L.H. Yang & M. Kang sp. nov.

urn:lsid:ipni.org:names:77175494-1

Figures 1 , 2

#### Diagnosis.

*Primulina malipoensis* mainly differs from *P. maguanensis* and *P. lungzhouensis* by its pale greenish-yellow flowers (vs. purple, with different colour patterns). This new species can further be distinguished from *P. maguanensis* by its greenish bracts (vs. white) and from *P. lungzhouensis* by its entire bracts margin (vs. denticulate).

#### Type.

CHINA. Guangdong Province, Guangzhou City, voucher from a cultivated plant at South China Botanical Garden, 29 July 2016 (flowering), *Li-Hua Yang*, *YLH369* (holotype: IBSC!), introduced from Yunnan province, Malipo county, Xiajinchang town, growing on moist limestone rocks, Alt. 1500 m, 23°10'N, 104°49'E, 31 August 2013, *Jun-lin Chen*.

#### Description.

*Perennial herbs*. *Rhizomatous* stem subterete, 20–60 mm long, 5–15 mm in diameter. *Leaves* 8–12, basal or clustered at apex of stem, opposite decussate. *Petiole* flattened, 20–40 mm long, 8–10 mm wide, pubescent. *Leaf blade* slightly fleshy when fresh, thickly chartaceous when dried, ovate to broadly elliptic, 7–12 × 7–10 cm, adaxially densely pubescent, abaxially glabrescent and only puberulent along veins, apex subacute to obtuse, base cuneate, margin inconspicuously serrate; *lateral veins* 4 on each side, abaxially conspicuous. *Cymes* 3–5, axillary, 2–4 branched, 8–16-flowered; *peduncles* 15–27 cm long, ca. 2 mm in diameter, densely pubescent; *bracts* 2, sometimes with bracteoles (narrowly ovate, 8–12 × 4–7 mm), green, opposite, ovate, 16–25 × 8–13 mm, margin entire, apex acute, outside densely pubescent, inside sparsely pubescent. *Pedicel* 10–14 mm long, ca. 1 mm in diameter, densely glandular pubescent and puberulent. *Calyx* 5-parted to near base, lobes narrowly lanceolate, white, 7–9 × ca. 2 mm, outside densely glandular pubescent and puberulent, inside sparsely pubescent, margin entire. *Corolla* pale greenish-yellow, 24–32 mm long, outside glandular-pubescent and puberulent, inside glabrous; *tube* infundibuliform, 21–25 mm long, ca. 8 mm in diameter at mouth, ca. 5 mm in diameter at base; *limb* distinctly 2-lipped, adaxial lip 2-parted, lobes broadly ovate, 7–9 × 6–7 mm, apex rounded, abaxial lip 3-lobed, lobes oblong, 11–13 × 5–7 mm, apex rounded. *Stamens* 2, adnate to 10–13 mm above the corolla tube base; *filaments* linear, 9–11 mm long, pale greenish-yellow, geniculate near middle, sparely pubescent; *anthers* fused by the entire adaxial surfaces, ca. 2 mm long, abaxially densely covered with glandular hairs. *Staminodes* 3, lateral ones 6–7 mm long, adnate to 10–12 mm above the corolla tube base, middle one ca. 1.5 mm long, adnate to 6–8 mm above the corolla tube base. *Disc* annular, ca. 1.5 mm in height. *Pistil* 22–26 mm long; *ovary* cylindrical, 15–18 mm long, ca. 1.5 mm in diameter, densely glandular pubescent and puberulent; *style* ca. 7 mm long, densely glandular-pubescent and puberulent; *stigma* 1, its upper lobe lacking, lower lobe obtrapeziform, shallowly 2-lobed at apex, ca. 2 mm long, ca. 1.5 mm wide. *Capsule* linear, ca. 30 mm long, densely pubescent.

**Figure 1.**
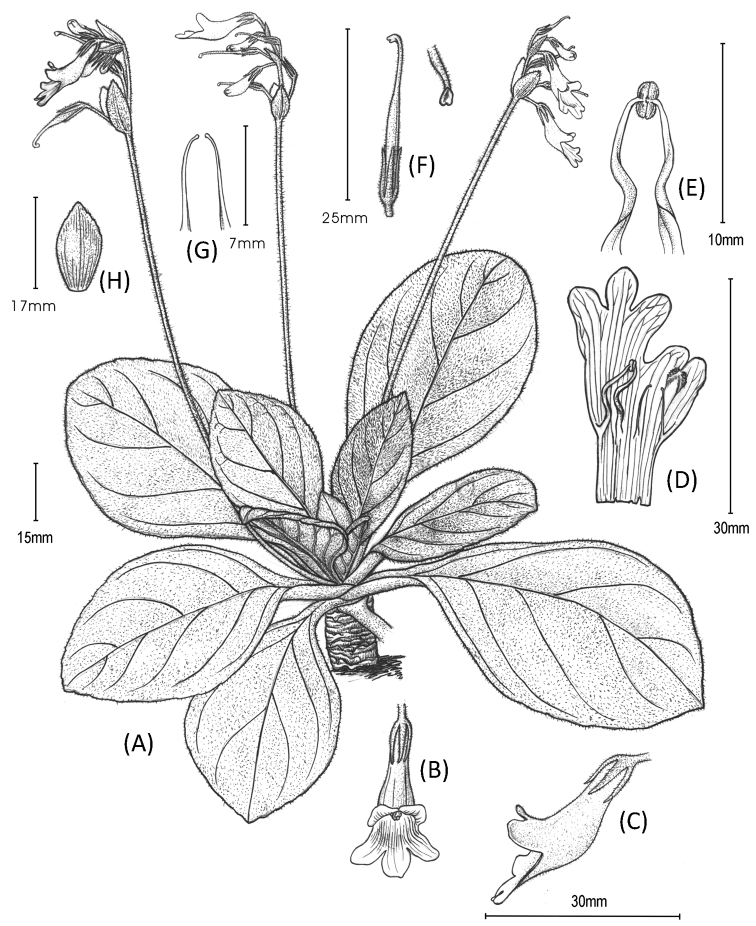
*Primulina malipoensis*. **A** habit **B** flower in front view **C** flower in side view **D** opened corolla, showing stamens and staminodes **E** fertile stamens **F** pistil and stigma **G** staminodes **H** bract. Drawn by Yun-Xiao Liu based on a cultivated individual collected from type locality.

**Figure 2.**
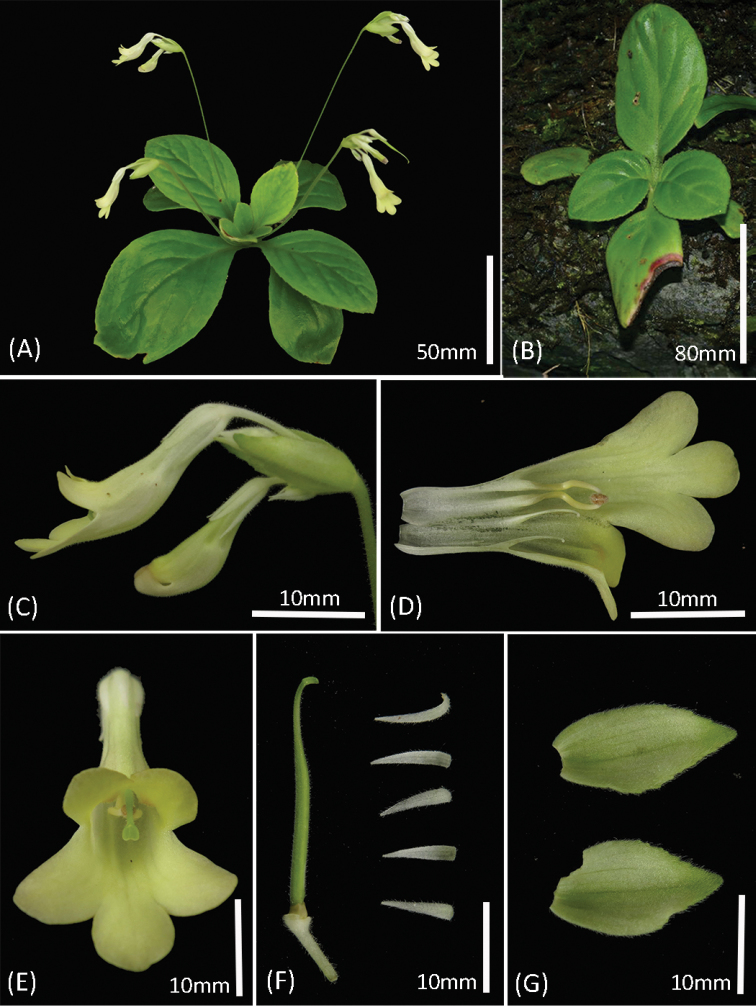
*Primulina malipoensis*. **A** flowering plant cultivated in South China Botanical Garden **B** plant in natural habitat **C** flower in side view **D** opened corolla, showing stamens and staminodes **E** flower in front view **F** pistil and calyx **G** bracts. Photographs by Li-Hua Yang.

**Figure 3.**
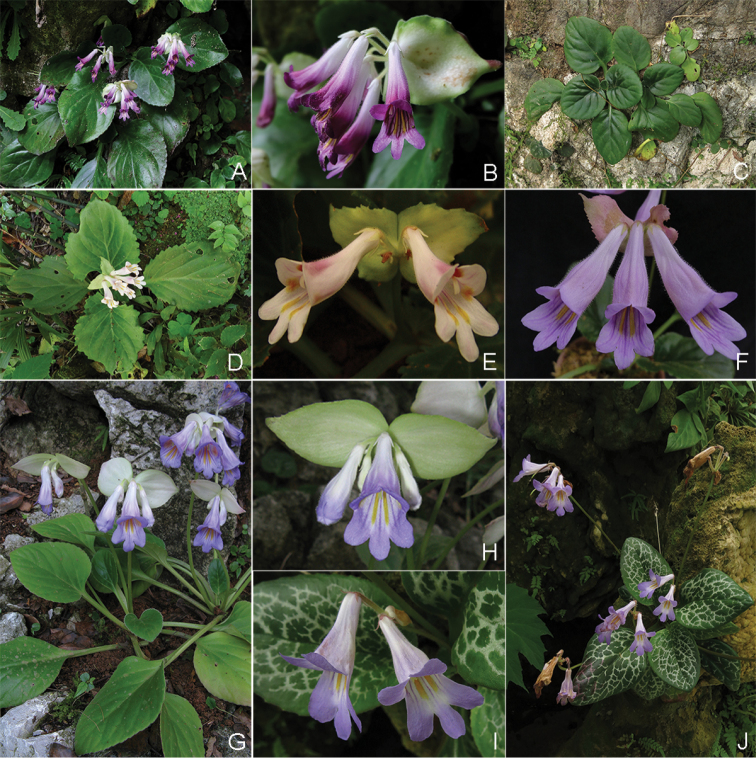
*Primulina maguanensis* (**A, B**), *P. lungzhouensis* (**D, E**), P. beiliuensis var. fimbribracteata (**C, F**), P. beiliuensis var. beiliuensis (**G, H**) and *P. maculata* (**I, J**). (**A, C, D, G, J**) habit, (**B, E, F, H, I**) flower. Photographs by Fang Wen (**A–H**) and Li-Hua Yang (**I, J**).

#### Distribution and habitat.

**Figure 4.**
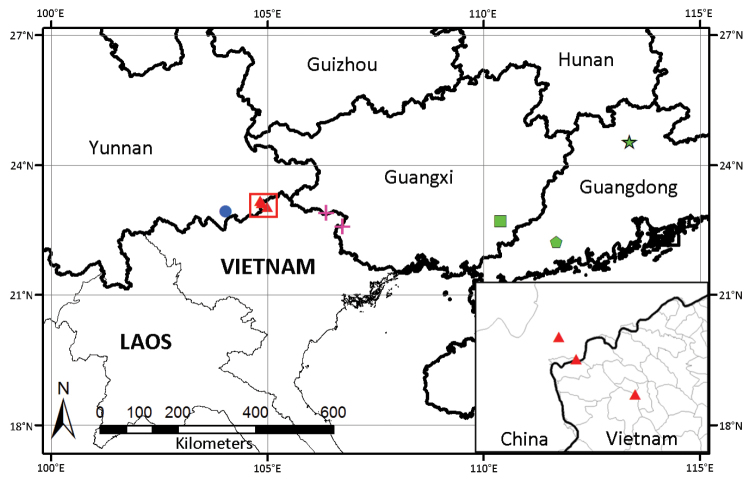
Geographical distribution of *Primulina malipoensis* (triangle), *P. lungzhouensis* (cross), *P. maguanensis* (dot), *P. maculata* (pentagon), P. beiliuensis var. beiliuensis (square) and P. beiliuensis var. fimbribracteata (star).

*Primulina malipoensis* is a narrowly endemic species restricted to a small area at both sides of the Sino-Vietnamese border (Xiajinchang Town, Malipo County, Yunnan Province, China. Khau La Village, Quyet Tien Community, Quan Ba District, Ha Qiang province, Vietnam.) (Figure 4). It grows on moist and shady limestone rocks, at ca. 1000–1500 m altitude.

#### Conservation status.

Based on the field investigations, *Primulina malipoensis* is currently only known from three sites around the Sino-Vietnamese boundary. Each population possesses no more than 150 mature individuals. However, the type population, which grew close to a road, had disappeared in 2017 and thus, the primary reason why it disappeared is probably due to its destruction by human activities. Based on currently available information, *P. malipoensis* should be considered as Endangered (EN): B1b(iii,v)c(iv)+2b(iii,v)c(iv); C2b, following the IUCN Categories and Criteria (IUCN 2016).

#### Phenology.

This new species was observed flowering from June to July and fruiting from August to September.

#### Etymology.

The specific epithet is derived from the place, Malipo County in Yunnan province, China, where the new species was first found.

#### Note.

*Primulina malipoensis* (Figures 1 and 2) can be morphologically connected to *P. maguanensis* (Z. Yu Li, H. Jiang & H. Xu) Mich. Möller & A. Weber (Figure 3A–B) and *P. lungzhouensis* (W.T. Wang) Mich. Möller & A. Weber (Figure 3D–E) by its ovate or broadly elliptic leaf blade, with inconspicuously (or conspicuously) serrate margin, obvious bracts, white calyx lobes and infundibuliform corolla tube. However, it can easily be distinguished from the latter two species by the characters summarised in the diagnosis.

The authors’ molecular phylogenetic analyses illustrate that *P. malipoensis*, *P. lungzhouensis*, *P. beiliuensis* B. Pan & S.X. Huang (Pan et al. 2013) and P. beiliuensis B. Pan & S.X. Huang var. fimbribracteata. F. Wen & B.D. Lai (Lai and Wen 2015) form a monophyletic clade (Kong et al. 2017). However, their morphology and geographical distribution allow the assumption that *P. maguanensis* and *P. maculata* W.B. Xu & J. Guo (Guo et al. 2015) are also closely related to this group. Both *P. maguanensis* and *P. maculata* were compared to *P. eburnea* in the original protologue (Xu et al. 2008, Guo et al. 2015). Nevertheless, based on the observation of living plants, *P. maguanensis* seems most similar to *P. lungzhouensis* and *P. malipoensis*; *P. maculata* (Figure 3I–J) seems most similar to P. beiliuensis var. beiliuensis (Figure 3G–H) and P. beiliuensis var. fimbribracteata (Figure 3C–F). Further, the geographical distribution of *P. maguanensis* is adjacent to *P. lungzhouensis* and *P. malipoensis* (Figure 4) and the geographical distribution of *P. maculata* is adjacent to *P. beiliuensis* (Figure 4). Moreover, the results of the phylogenetical analysis in Guo et al. (2015) show that *P. maculata* is more closely related to *P. lungzhouensis* than *P. eburnea*. All of the above five species occur in nearly the same latitude zone of karst limestone areas from Southern China (from S-Yunnan to S-Guangdong), but with a disjunctive distribution (Figure 4). Therefore, these species perhaps represents a complex of longitudinal speciation, which may be caused by geographical isolation. Further studies are needed to confirm the phylogenetic relationship of this species complex and to determine its evolutionary mechanism of speciation.

*Primulina malipoensis* could also be related to other species by its yellow flowers. However, the phylogenetic results illustrate that *P. malipoensis* has a distant relationship with all yellow flowering species, such as *P. lutea* (Yan Liu & Y. G. Wei) Mich. Möller & A. Weber, *P. alutacea* F. Wen, B. Pan & B.M. Wang (Pan & al. 2016), *P. pteropoda* (W.T. Wang) Yan Liu, *P. leprosa* (Yan Liu & W.B. Xu) W.B. Xu & K.F. Chung and *P. jiangyongensis* X.L. Yu & Ming Li (Li et al. 2014) (cf. Kong et al. 2017). These yellow flowering species are distributed across different clades (Kong et al. 2017), which means that yellow flowers have independently evolved in different species. This result also suggests that flower colour can be used as an important character to differentiate species in *Primulina*.

#### Other specimen examined.

CHINA. Yunnan Province, Malipo county, Xiajinchang town, Aotang, 23°07'45.41"N, 104°51'29.25"E, Alt. 1400 m, growing on moist limestone rocks near a road, 8 January 2010, *Southeast Yunnan investigation team of DNA barcoding*, *GBOWS189* (KUN!). CHINA. Guangdong Province, Guangzhou City, voucher from a cultivated plant at South China Botanical Garden, 12 June 2016 (flowering), *Li-Hua Yang*, *YLH350* (IBSC!), introduced from same locality and by the same people as the type. VIETNAM. Ha Qiang province, Quan Ba District, Quyet Tien Community, Khau La Village, Alt. 1100 m, growing on moist limestone rocks, 17 October 2017, *Fang Wen et al. VMN-CN 874* (IBK!, VMN!).

## Supplementary Material

XML Treatment for Primulina malipoensis
